# Pregnancy Associated with Systemic Lupus Erythematosus: Immune Tolerance in Pregnancy and Its Deficiency in Systemic Lupus Erythematosus—An Immunological Dilemma

**DOI:** 10.1155/2015/241547

**Published:** 2015-05-18

**Authors:** Cristina Gluhovschi, Gheorghe Gluhovschi, Ligia Petrica, Silvia Velciov, Adrian Gluhovschi

**Affiliations:** ^1^Division of Nephrology, University of Medicine and Pharmacy “V. Babes” Timisoara, Calea Alexandru Ioan Cuza No. 8 Ap. 16, 300088 Timisoara, Romania; ^2^Division of Nephrology, Emergency County Clinical Hospital Timisoara, Romanian Academy of Medical Sciences, Romania; ^3^Division of Nephrology, University of Medicine and Pharmacy “V. Babes” Timisoara, Emergency County Clinical Hospital Timisoara, Romania; ^4^Department of Obstetrics and Gynecology, University of Medicine and Pharmacy “V. Babes” Timisoara, Emergency County Clinical Hospital Timisoara, Romania

## Abstract

Pregnancy is a physiological condition that requires immune tolerance to the product of conception. Systemic lupus erythematosus (SLE) is a disease with well-represented immune mechanisms that disturb immune tolerance. The association of pregnancy with systemic lupus erythematosus creates a particular immune environment in which the immune tolerance specific of pregnancy is required to coexist with alterations of the immune system caused by SLE. The main role is played by T regulatory (Treg) cells, which attempt to regulate and adapt the immune system of the mother to the new conditions of pregnancy. Other components of the immune system also participate to maintain maternal-fetal immune tolerance. If the immune system of pregnant women with SLE is not able to maintain maternal immune tolerance to the fetus, pregnancy complications (miscarriage, fetal hypotrophy, and preterm birth) or maternal complications (preeclampsia or activation of SLE, especially in conditions of lupus nephritis) may occur. In certain situations this can be responsible for neonatal lupus. At the same time, it must be noted that during pregnancy, the immune system is able to achieve immune tolerance while maintaining the anti-infectious immune capacity of the mother. Immunological monitoring of pregnancy during SLE, as well as of the mother's disease, is required. It is important to understand immune tolerance to grafts in transplant pathology.

## 1. Introduction

The association of systemic lupus erythematosus (SLE) with pregnancy represents a particular situation in immunopathology.

This is closely related to specific immune changes of the maternal body during pregnancy that ensure immune tolerance to the product of conception which presents paternal antigens and therefore represents a semiallogeneic graft for the host. In fact, pregnancy is considered “a major challenge to the maternal immune system” [1].

Important immune alterations occur in patients with SLE, including deficiencies of the immune system as well as immune tolerance.

The association of pregnancy with a modified immune system adapted to immune tolerance to fetal antigens with a disease with a strongly impaired immune system, with deficiencies concerning immune tolerance mechanisms, represents an entirely special aspect in immunopathology.

The cornerstone of the relationship between the immune system in pregnancy and the immune system in SLE is represented by T regulatory (Treg) cells. Pregnancy-related hormonal changes such as hyperestrogenism are added to this relationship, and the immune cells are sensitive to these changes. One can also observe a relationship between the immune system and hormonal factors, mainly estrogens, among patients with SLE.

In cases of pregnancy associated with lupus erythematosus, important interrelations occur between the immune system of the mother and the immune system of the fetus. Alterations in immune mechanisms can have severe consequences both for the fetus, including a risk of miscarriage or disease transmission (neonatal lupus), and for the mother, including activation of SLE.

The aim of this paper is to present the interrelationship between the immune mechanisms in pregnancy and the immune mechanisms in SLE in cases of pregnancy associated with lupus erythematosus. Although many elements remain unknown, we consider an updated presentation useful.

## 2. Specifics of Immunology of Pregnancy

From an immunological point of view, pregnancy is an allograft with the following particularities.

- The fetus has 50% paternal antigens.
- The fetus is separated from the mother by a maternal-fetal interface. Among the components of this interface, we distinguish the trophoblast, which represents a cellular layer that does not allow contact between fetal antigens and maternal antigens.
- The specific hormonal environment is represented by high levels of estrogens and progesterone.

In this situation, the maternal immune system has to achieve conditions of immune tolerance, while also maintaining its anti-infectious capacity.

This characteristic of the immune system, which on the one hand ensures immune tolerance and on the other hand maintains reactivity against pathogens, demonstrates its particular adaptability.

The maternal immune system ensures antibacterial activity mainly by means of antibodies. Bacterial antigens are taken up by antigen-presenting cells. Stimulation of B cells occurs with production of antibodies. T helper cells participate as costimulatory cells.

A shift at the Th 1 and Th 2 helper cell level occurs. Th 2 cells dominate in pregnancy and also suppress the response of cytotoxic T cells. The Th 1-Th 2 shift leads to suppression of antifetal antigen-mediated immune responses.

The hormonal system participates in the suppression of cell-mediated immunity, and thus immune tolerance.

A tight cooperation for preventing a response to fetal antigens occurs between the trophoblast and the maternal immune system.

According to Mor and Cardenas, immune mechanisms in pregnancy combine a “signal of response of the maternal immune system and fetal-placental immune system.” They suggest that the fetal-placental immune system could play an important mediating role for the maternal immune system [2].

## 3. SLE

SLE is an autoimmune disease that predominantly affects women during their reproductive years.

The intervention of hormonal factors is involved in SLE. Alterations in estrogen metabolism occur, with high levels of 16-alpha-hydroxyestrone. The estrogen receptor and its relationship with cytokines are very important [3]. Additionally, the level of androgens diminishes.

Increased estrogens influence the immune system. The proliferation of B-cells and the production of antibodies increase, influencing the proliferation of T cells. Prolactin, an immunostimulatory hormone, is also affected, as is the gonadotropin-releasing hormone, the hypothalamus-pituitary axis being defective in SLE.

Humoral immune mechanisms are impaired, with formation and deposition of immune complexes and components of the complement system in tissues.

Apoptosis leads to elimination of molecules from the nucleus, the cytoplasm, and the cell surface. The clearance of these products is deficient [4]. The products are taken up by macrophages and presented to T cells, subsequently activating them. Dendritic cells are also involved as antigen-presenting cells.

Autoantibodies are produced as a consequence of B-cell activation. The most representative are antinuclear antibodies. Anti-double-stranded DNA (ds-DNA) and anti-Sm antibodies are specific for SLE.

## 4. Dysregulation of the Immune Response

Immune system abnormalities involve B cells, T cells, and monocytes, with polyclonal B activation and increased autoantibody production.

In SLE, alterations of the immune system affect T regulatory cells, which seem to play an important role in the dysfunction of the immune system.

## 5. Pregnancy and Systemic Lupus Erythematosus

The function of the immune system as a whole is altered in SLE, which results in alteration of immune tolerance not only to self-antigens. Pregnancy is founded on tolerance of the maternal immune system to paternal antigens present in the fetus.

In cases of pregnancy associated with SLE, the main immune alteration involves the function of Treg cells [5].

In SLE, the number of Treg cells and their functions are limited. However, Treg cells have an important role in regulating the immune system and in maintaining self-tolerance. In pregnancy, immune tolerance to the fetus is mainly ensured by Treg cells. In cases of pregnancy associated with SLE, the immune system is confronted with the dilemma of how to adapt to ensure favorable development of the product of conception. How does tolerance to fetal antigens occur in the presence of a disease with altered tolerance to self-antigens?

The relationship between pregnancy and SLE is even more complex because estrogens are involved in the immunopathology of SLE. Pregnancy also presents an immune system under the influence of increased estrogen levels. Other hormonal factors, including progesterone and prolactin, are also involved.

It should be noted that numerous factors of the immune system that will be presented here play an important role in shaping the immune tolerance in cases of pregnancy associated with SLE. We specifically mention that the presence of maternal antiphospholipid antibodies can adversely affect pregnancy. Additionally, maternal anti-Ro and anti-La antibodies can cross the placental barrier and can cause neonatal lupus.

It is worth mentioning that in cases of SLE, the loss of immune tolerance to the fetus can have both fetal (miscarriage, intrauterine growth restriction (IUGR), preterm birth, and neonatal lupus) and maternal (preeclampsia and activation of SLE) consequences [6].

Our paper aims to analyze the main immune factors that participate in cases of SLE associated with pregnancy.

## 6. T Regulatory Cells (Treg Cells)

One of the functions of T helper cells is to modulate the immune response. Their function is regulated by T regulatory cells (Treg cells). According to Saito et al., there is a new paradigm of T cells, namely, Th 1/Th 2/Th 17, which is related to Treg cells. Treg cells, by means of immunomodulatory cytokines such as TGF-beta, can suppress the capacity of these cells to produce cytokines [7].

Two types of T regulatory cells are described; some originate at the level of the thymus (natural Treg cells), while others are induced in the periphery (induced Treg cells).

Their role is to control effector functions of immune cells such as macrophages, cytotoxic CD8 cells, and NK cells. They also have a role in regulating immune responses, and they are involved in cytokine secretion (IL 10, TGF-beta, etc.).

Treg cells bear the CD25 antigen, which is a marker of their activation [5].

Adaptive Treg cells have the following subtypes: IL 10-producing Trl cells, Th 3 cells (TGF-producing Treg cells), and CD4^+^ CD25^+^ Foxp3 Treg cells [8].

CD4^+^ CD25^+^ Treg cells suppress the potential action of autoreactive cells [9].

CD4^+^ CD25^+^ Treg cells express a protein, Foxp3, which is a marker of their activation [9]. Another marker of Treg cells is CTLA-4, which is associated with suppressive functions.

After stimulation and activation, Treg cells are susceptible to action. T cells originating in the thymus can intervene to protect against Th 1-mediated responses to autoantigens [10].

Treg cells accumulate in the decidua. At the same time, they are numerous in maternal blood during the first trimester.

Treg cells act at the maternal-fetal interface. At this level, they regulate immune cell responses by interacting with other cells and regulating the expression of immune regulatory molecules [11].

According to Aluvihare et al., maternal Treg cells suppress an aggressive allogeneic response directed against the fetus. Their absence could impair the continuation of pregnancy by resulting in immune rejection of the fetus [1].

Abnormalities of CD4^+^ CD25^+^ Foxp3 Treg cells could contribute to T- and B-cell hyperactivity in SLE [12].

The number of Treg cells increases in normal pregnancy and decreases in cases of pregnancy loss and preeclampsia [11].

The decrease in the number and functionality of Treg cells can predispose women with systemic lupus erythematosus to pregnancy complications [13].

Pregnancy associated with SLE represents a special situation for Treg cells. On the one hand, pregnancy benefits from the contribution of Treg cells, which ensure maternal-fetal tolerance. On the other hand, Treg cells are defective in SLE.

In cases of pregnancy associated with inactive SLE, Treg cells might ensure maternal-fetal tolerance because functional Treg cells predominate.

It is also possible that in cases of pregnancy associated with SLE, inactive Treg cells impair maintenance of fetal immune tolerance and result in complications such as miscarriage, preterm birth, or preeclampsia [1].

Tower suggests that women with SLE have dysfunctional tolerance capabilities that compromise their adaptability to pregnancy [11].

This is why women with SLE are dissuaded from becoming pregnant prior to remission of SLE.

## 7. Th 17 Cells

T helper cells are regulated by Treg cells. The Th 17 cell subset mainly produces IL 17. Other interleukins that are produced by these cells include IL 21, IL 22, and IL 17F.

Th 17 cells play a protective role against pathogenic germs and are central to the relationship between the self-antigens of inflammation and those of autoimmunity [14]. Th 17 cells have a role in inflammatory processes in autoimmune diseases [15].

Th 17 cells intervene in recurrent pregnancy loss and preeclampsia [16]. Changes in the ratio of Th 17 cells to Treg cells may be related to spontaneous abortion and premature birth [7].

Th 17 cells possess great plasticity. Cells which mainly produce IL 17A and IL 17F can turn into cells that produce interferon gamma [17].

Torricelli et al. found that pregnant women with SLE demonstrated increased levels of IL 17 together with other cytokines, including IL 6, IL 10, and TNF. This may indicate a hyperactive immune system among pregnant women with SLE, and this may be related to the placenta [18].

Estrogens play a role in Th cell secretion of IL 17, as demonstrated in mice [19]. They inhibit the secretion of Th 17 [20]. Estrogen levels are high during pregnancy, and patients with unexplained recurrent abortion have high levels of Th 17 cells in their blood and decidua [21].

Nakashima et al. found a high number of IL 17-positive cells in the decidua of abortion cases and suggested that Th 17 cells could be involved in the induction of inflammation in the late stage of abortion [22].

By promoting Th 2 responses, estrogens in pregnancy tend to worsen Th 2-mediated diseases such as SLE [23]. Torricelli showed high levels of serum IL 17 in pregnant women with SLE [18].

Th 17 and estrogens are involved in processes of osteogenesis. Th 17 cells are considered to be “a new candidate in the pathogenesis of osteoporosis.” Estrogen deficiency was found to play a role in inducing the differentiation of IL 17-secreting Th 17 cells [20].

Progesterone is involved in autoimmune processes. Its action consists in the suppression of Th 17- and Th 1-related responses [24].

In pregnancy, progesterone contributes to a favorable environment for pregnancy through its favorable effects on Th 2 and Treg cells and its suppression of Th 1 and Th 17 cells [21].

The immune response to antigens such as candida albicans via Th 17 cytokines is diminished in the presence of high levels of estradiol, while progesterone associated with lower estradiol levels restore the pathogen-host equilibrium, a fact demonstrated by experimental studies in mice [25].

## 8. B Cells

B cells have an important role in producing antibodies. In SLE, they produce autoantibodies, which can play a pathogenic role.

According to Fettke et al., B cells participate in maternal immune tolerance to the fetus, with IL 10, a modulating cytokine produced by B cells, playing an important role [26]. These cells (B10) are a subset of B2 cells called regulatory B cells. Thus, B cells play an adaptive role. It was found that maternal lymphocytes specific for the paternal histocompatibility antigens undergo a process of partial deletion. The important role played by B cells could be reflected in the success of the fetal allograft [27].

According to Fettke et al., aberrant B cell behavior is related to obstetric pathology [26].

In SLE, B cells participate in the production of antibodies, which play an important role in the initiation, evolution, and exacerbation of the disease. This fact led to the use of rituximab in SLE therapy, the depletion of B cells with rituximab being accompanied by a decrease in autoantibody production [28].

The control of B cell proliferation depends strictly on Treg cells. In SLE, the control of autoantibody production is lost, but regulatory T cells play an important role in SLE and pregnancy [11]. Muzzio et al. consider “the role of B cells in pregnancy: the good and the bad.” A balance between activation and tolerance is present. B cells participate in the normal evolution of pregnancy and in pregnancy-associated pathology [29].

Although therapeutic modulation with rituximab has proven to be effective in SLE, its use during pregnancy is not allowed.

It is possible that future risk-free immunomodulatory medications may be developed for SLE that target B cells and allow for favorable pregnancy development [30].

## 9. Autoantibodies

In SLE, impaired removal of apoptotic cellular material occurs due to deficient clearance. This leads to the production by B cells of antibodies against these structures, which are referred to as autoantibodies. These autoantibodies are common in SLE and vary according to the structures affected by SLE.

The most important autoantibodies in SLE are those against nuclear structures, referred to as antinuclear antibodies. They are used in the diagnosis of SLE.

Anti-double-stranded DNA antibodies are very specific for SLE. Anti-single-stranded DNA antibodies and anti-RNA antibodies can also be present in SLE.

Anti-Sm antibodies are increasingly found during renal involvement in SLE [31]. Anti-Ro and anti-La antinuclear antibodies are very important in pregnancy. They can cross the placenta and can induce fetal injury, causing neonatal lupus [32].

Some autoantibodies have organ specificity. For example, anti-N-methyl-D-aspartame (NMDA) antibodies are present in central nervous system lupus.

Autoantibodies against red blood cells (producing hemolytic anemia), as well as antiplatelet antibodies, have been described.

The diversity of antibodies in SLE can be explained by the complexity of immune mechanisms.

Pregnancy imposes immune tolerance to paternal antigens. However, SLE triggers immune reactions with cytotoxic characteristics. In this situation, the protective maternal immune mechanisms developed during pregnancy compete with the cytotoxic characteristics. The variety of autoantibodies present in SLE can also influence pregnancy outcome.

Thus, antiphospholipid antibodies can play a detrimental role in the development of pregnancy, as can the abovementioned anti-Ro and anti-La antibodies, which can induce neonatal lupus.

However, it is recommended that women with SLE do not become pregnant during the 6-month period following an SLE flare-up, as autoantibody titers are low when the disease is inactive.

## 10. Antiphospholipid Antibodies

Antiphospholipid antibodies (aPLs) are associated with SLE and other autoimmune diseases. They can have fetal and maternal consequences during pregnancy.

Antiphospholipid antibodies are associated with miscarriage, fetal death, intrauterine growth restriction (IUGR), preterm birth, and preeclampsia [33], and an association with recurrent miscarriage has also been reported [34].

Antiphospholipid syndrome is frequently associated with the HELLP syndrome, an association mentioned by Le Thi Thuong et al. [35]. Placental insufficiency can occur in the presence of aPLs [36].

The importance of aPLs was shown in a study of McNeil et al., who reported fetal demise in 38–59% of pregnancies associated with SLE, compared to 16–20% of pregnancies without these antibodies [37].

Maternal and fetal complications are considered to be related at least partly with the presence of aPLs [38].

Immunoglobulin, heparin, and small doses of aspirin are used to treat the anticardiolipin syndrome [39].

## 11. Innate Lymphoid Cells (ILC)

A new type of cells involved in innate immunity, the ILC cells, has recently been defined. They are not homogenous, and they consist of 3 main groups. Group 1 contains NK cells, group 2 contains ILC 2 cells, and group 3 contains ILC 3 cells and a subgroup defined as lymphoid tissue inducer cells (LTi).

ILC cells play a distinct part in innate immune responses associated with the production of Th 1, Th 2, and Th 17 cytokines [40].

*Group 1* is related to the intervention of NK cells in pregnancy and in SLE.

*NK cells* are considered to be a “founding member of the innate lymphoid cell family” [41]. Thus, NK cells intervene in maintaining homeostasis, secreting both protective and proinflammatory cytokines [42].

NK cells participate in immune regulation processes by playing a role in regulating production of antibodies that are dependent on T cells involved in autoimmune disease [43]. NK cells also participate in host defense and play an important role in infections.

NK cells have an important role both in fertility and in development of pregnancy, and they also play an important role in implantation [43].

According to Baxter and Smyth, NK cell populations are lower among patients with SLE than in controls [44].

Regarding the serum values of NK cells, Su et al. found no difference between the number of NK cells in pregnant women with and without SLE [45].

In SLE, there is a reduction in NK activity, which decreases markedly in severe cases and in cases of lupus nephritis [44].

There are higher concentrations of decidual NK cells in complicated pregnancies and in cases of recurrent miscarriage; treatment with glucocorticoids is currently being discussed [46].

Decidual cells, which are located at the fetal-maternal interface, have the capacity to produce cytokines, including IL 1B and TGF. They can participate in autoimmune processes at this level via these cytokines [47]. During pregnancy, NK cells are in close contact with the fetal interface. NK cells in the decidua have limited activity compared to NK cells in the trophoblast and in the blood [48].

NK cells are found at decidual level and around spiral arteries. They play a role in modulating trophoblast invasion and vascular remodeling, and they are interrelated with cells at the level of the decidua. This relationship is impaired in preeclampsia. In fact, it is generally known that uterine NK cells intervene in the development of normal pregnancy [49].

Wallace et al. noticed that NK cell receptor expression is altered in pregnancies at higher risk of preeclampsia [50].

According to Pereira et al., while the innate immune system is active in pregnancy, there is suppression of adaptive immunity [43].

NK cells can also regulate the target cells by apoptosis.

Total apoptosis in SLE is low. NK cells have a prolonged life cycle, which could represent a higher immune stimulus for activating apoptotic processes [43].

*Group 2*, consists of Th 2-type innate lymphocytes that produce the following type 2 cytokines: IL 5 and IL 13 [51].

Group 2 cells are found in the human respiratory and gastrointestinal tract, in the skin and in the spleen. ILC 2 cells intervene in antiparasite immunity, allergy, asthma, and atopic dermatitis.

Classic NK cells and Th 2 type ILC cells intervene in the production of Th 1, Th 2, and Th 17 cytokines [40].

There are no studies on the intervention of ILC 2 in pregnancy and SLE.

*Group 3* (innate lymphoid cells 3/ILC3) are present at the level of the mucosa, at the level of Peyer's patches and gut-associated lymphoid tissue (GALT). Small quantities are found in the spleen and in the lungs.

A subset of ILC 3 cells is represented by lymphoid tissue inducer (LTi cells), which produce IL 17 and IL 22. The profile of LTi cells is similar to that of Th17 cells.

These cells are involved in the development of lymphoid nodes and Peyer's patches, which are programmed during the fetal period [52].

They ensure the relationship between ILC and NK cells, facilitating via a lymphotoxin NK cell development.

Until recently, only NK cells have been identified and functionally characterized at the level of the human decidua. However, in 2014, Vacca et al. identified two subsets of the ILC 3 decidual group. One of these subsets produces IL 17 and TNF, and the other produces IL 2a and IL 8 [53].

They could contribute to innate defense and tissue building, thus playing a part in the continuation of pregnancy.

ILC cells require further study to elucidate their relationship with the immune system in pregnancy.

## 12. Gamma/Delta Cells

Numerous gamma/delta cells are located at the level of the maternal-fetal interface [54]. According to Szekeres-Bartho et al., HLA G presents antigens to gamma/delta cells. At the same time, gamma/delta cells can recognize unprocessed foreign antigens without the MHC [55].

The response of lymphocytes that bear gamma/delta receptors is related to progesterone, being considered as progesterone-dependent immunomodulation [56].

According to Su et al., gamma/delta cells play a role in immune responses being involved in autoimmune diseases [57].

Fujii et al. identified in lupus-prone mice a gamma/delta cells line that suppresses autoantibody synthesis [58].

## 13. Monocytes/Macrophages

During pregnancy, peripheral monocytes undergo adaptive phenomena. They consist of decreased expression of chemokine receptors, as well as alteration of the response to microbial stimuli. The trophoblast cells secrete chemokines and recruit monocytes [59].

Björkander et al. suggest that the expression of monocyte chemokine receptors creates a unique chemokine milieu during pregnancy. In SLE, there is increased expression of CCR5 on CD16^+^ monocytes [59].

Decidual macrophages express inhibitory receptors that can bind to HLA-G, being expressed on invading extravillous trophoblasts.

Afterwards, transmission of a negative signal can block their action resulting in immune tolerance and induction of anti-inflammatory cytokines [60].

## 14. Dendritic Cells

Palucka et al. reported alterations in immune cell homeostasis that involve dendritic cells. SLE is considered to present continuous activation of myeloid dendritic cells produced by plasmacytoid dendritic cells via alpha interferon [61].

Dendritic cells are involved in controlling immunity and immune tolerance, and dendritic cells play an important role at the level of the maternal-fetal interface [62].

In SLE, maturation of myeloid dendritic cells secondary to deficient apoptosis will cause induction of Th 17 cells with production of IL 17 [63].

During pregnancy, myeloid dendritic cells induce differentiation of Th 1 cells, and plasmacytoid cells induce differentiation of Th 2 cells. T regulatory cells have suppressive activity.

In SLE, the ratio of Treg cells to dendritic cells maintains maternal tolerance. This alteration could be the basis of pregnancy complications associated with SLE [64].

## 15. Estrogens and Progesterone in the Immune Responses of Pregnancy and SLE

Estrogens are related to the immune system. Immune cells possess estrogen receptors. According to Tanriverdi et al., they are expressed in primary lymphoid organs and peripheral immune cells, and they can also play an active role in immune diseases [65]. Estrogens are considered to play an immunomodulatory role [66].

SLE is under the influence of estrogens. SLE predominantly affects women during their reproductive years, and there can be an increase in some active estrogen metabolites in this disease. During pregnancy, high levels of estrogens can be related to the Th 1/Th 2 ratio, which alters in favor of Th2. In cases of associations with SLE, there are consequences for the evolution of pregnancy. In SLE, estradiol levels decrease during the third trimester of pregnancy secondary to placental impairment. As a consequence of this process, the immune response, as well as SLE activity, decreases [67].

Iaccarino et al. noted that polarization towards Th 2 of the immune response during pregnancy and SLE is markedly less than in normal pregnancies [68].

It should also be noted that 17-beta-estradiol influences the production of IgG anti-double-stranded-DNA antibodies [69].

There is also hormonal regulation of B cell function in SLE [70].

## 16. Progesterone

In the second part of gestation, progesterone inhibits Th1-type cytokine production and induces production of Th2 cytokines and IL 10, which stimulate the humoral immune response.

According to Zen et al., high progesterone levels at the fetal-maternal interface level could play a role in the favorable development of pregnancy [71].

Hughes and Choubey suggest a relationship between estrogen and progesterone in SLE, in which the balance between the two determines disease expression [72].

Other hormones, such as prolactin and gonadotropin, also play a role in modulating immune responses.

## 17. The Maternal-Fetal Interface

During pregnancy, there is a special relationship between the mother and the fetus, as the fetus contains 50% paternal antigens.

This requires a barrier between the mother and the fetus to separate the fetal cells, which contain paternal antigens that are alien to the mother, from her immune system.

The relationship between the mother and the fetus is ensured via the placenta, which offers the nutritive substances needed by the fetus. One of the placental layers consists of the trophoblast, which separates the immune system of the mother from the fetal antigens and thus allows the development of the fetus. The maternal-fetal interface involves both the anatomic barrier formed by the trophoblast and the local immune changes that play a role in maintaining the fetal allograft.

According to Du et al., there is permanent cross-talk between the mother and the fetus at this interface [73]. Immune cells are present at the maternal-fetal interface, and there are several types of chemokines with the following functions:

- selective leukocyte traffic at placental level,
- trophoblast invasion,
- placental angiogenesis,
- recruitment and instruction of immune cells that ensure preservation of an environment that is favorable to pregnancy [73].

Numerous immune cells are found at the level of the maternal-fetal interface, with Treg cells playing a leading role [5].

Chorionic gonadotropin produced by the trophoblast also plays a role.

As far as histocompatibility antigens are concerned, as opposed to the HLA Class I molecules which are present on most somatic cells and present peptide antigens to T cells, nonclassical HLA G and HLA E are expressed. The main HLA molecule with strong expression in pregnancy is HLA G. It is expressed only at placental level. HLA G could be essential in maintaining immune tolerance to fetal antigens [74].

Immune tolerance is achieved by the activity of HLA molecules that act as ligands for receptors of NK cells and of macrophages [74].

Placental changes in SLE can adversely affect fetal development. Antiphospholipid antibodies can alter the placental phospholipid membrane. This happens mainly when antiphospholipid antibodies or other procoagulant conditions are present [75].

Vascular impairment of the placenta is evidenced by fibrin deposits caused by impairment of coagulation processes in which procoagulant factors also participate. One can detect coagulant changes related to the antiphospholipid syndrome, consisting of vascular changes or coagulation abnormalities. These can lead to villous thrombosis. Infarctions and edema/swelling can also be observed at placental level [76].

The participation of immune processes in the production of placental injury during pregnancy and SLE is documented by the presence immunoglobulins IgG, IgA, IgM, and C_3_ complement deposits. DNA-anti-DNA deposits can also be detected in pregnancy and SLE. The volume of the placenta decreases along with the number and size of the villi. Placental insufficiency can also be observed [77].

## 18. Complications of SLE During Pregnancy

Complications can involve the fetus, the mother, or sometimes both. Both maternal and fetal complications associated with SLE are related to the participation of immune mechanisms.

Pregnancy is considered an allograft. At the moment in which immune mechanisms no longer ensure the mother's tolerance to the product of conception, miscarriage or premature delivery occurs.

Among women with SLE, fetal loss occurs in up to 50% of pregnancies, though the incidence is lower at present.

Normal pregnancies are associated with functional placentas. In SLE, placental insufficiency is common. As a consequence, insufficient development of pregnancy occurs, which may result in IUGR, fetal death, or stillbirth [78].

Antiphospholipid antibodies are among the factors involved in fetal complications, and they are frequently associated with fetal loss. They could be the main cause of spontaneous miscarriage and recurrent miscarriage [79].

Anti-DNA antibodies cross react with laminin, which participates in placental implantation. Anti-DNA antibodies inhibit trophoblast attachment and migration, with implications in recurrent pregnancy loss in SLE [80].

Thrombocytopenia could also be involved in pregnancy loss [81].

The risk of fetal loss is high in pregnancies associated with active SLE and with lupus nephritis.

The role of the T helper cells should be mentioned. Th 1-mediated response via the intervention of proinflammatory cytokines could participate in producing miscarriage. They could counteract the anti-inflammatory cytokines produced by Th 2, Th 17, and Treg cells [82].

## 19. Neonatal Lupus

Children born to mothers with antibodies against Ro/SSA and La/SSB can present skin, cardiac, and systemic abnormalities, which are classified as neonatal lupus erythematosus [83].

These autoantibodies can cross the placenta to cause lesions in the fetus [84].

The main fetal injuries produced by these autoantibodies involve the heart, producing congenital heart block.

These lesions could originate in the activity of anti-Ro/SSA and anti-La/SSB autoantibodies, which act on calcium channels related to their regulatory proteins. The conduction abnormalities and the inhibition of L-type calcium channels have been highlighted by experimental studies in the rat heart model [85].

According to Wisuthsarewong et al., injuries produced by maternal autoantibodies can impair atrial fetal cells where inflammatory lesions appear with subsequent fibrosis and scarring of the atrioventricular node, the sinus node, and the His fascicle [86].

Anti-U_1_ RNP autoantibodies can be involved in the production of skin lesions. In these situations, skin lesions are not associated with cardiac ones.

Anti-U_1_ RNP autoantibodies, according to Okawa-Takatsuji et al., can be the basis for endothelial cell-binding activity in patients with connective tissue disease [87].

Shahian et al. found anti-Ro/SSA and anti-La/SSB autoantibodies in 98% of mothers who had given birth to children with neonatal SLE; however, only 1%-2% of mothers with these antibodies have neonates with neonatal lupus [88].

Meyer also reports that congenital heart block is rare and is present in only 1% of neonates of pregnant women with anti-Ro/SSA or anti-La/SSB autoantibodies [89].

In general, skin, liver, and blood (thrombocytopenia) complications tend to regress spontaneously after 4–6 months [90].

## 20. Maternal Complications

### 20.1. Preeclampsia

It is one of the complications occurring in SLE that can worsen the disease. Preeclampsia occurs most frequently in cases of lupus nephritis in the presence of anticardiolipin antibodies, thrombocytopenia, diabetes mellitus, and so forth. Preeclampsia is also observed in pregnant women with a history of preeclampsia in previous pregnancies [91].

In preeclampsia, there is a generalized inflammatory reaction related to the secretion of inflammatory factors by the placenta, which results in activation of neutrophils, monocytes, and endothelial cells [60].

SLE is also a complex inflammatory condition. Immune changes are found in preeclampsia, the most important being the Th 1–Th 17/Th 2-Treg imbalance (Perez-Sepulveda et al). In SLE, there is an important alteration of the relationship between Th 1 and Th 2 cells [92].

*Lupus nephritis* presents a special condition in pathology when associated with pregnancy. In lupus nephritis, immune mechanisms mediated by immune complexes, mostly composed of anti-DNA antibodies, and elements of the complement system participate. Cellular immunity with alteration of Treg cell functions also plays an important role.

In cases of active disease, the newly formed complexes can lead to activation of lupus nephritis. This is why pregnancy is recommended only after lupus nephritis has been inactive for 6 months.

Active lupus nephritis is frequently associated with fetal complications of IUGR, preterm birth, and fetal death, as well as with maternal complications such as preeclampsia.

According to Fatemi et al., the absence of lupus nephritis is important to prevent SLE activation [93].

### 20.2. SLE Flares

SLE may develop before or during pregnancy. Pregnancy that is superimposed on active SLE can worsen the disease. According to Ruiz-Irastorza et al., flares are more frequent during the second and third trimester and during the postpartum period [94].

Women with SLE are counselled to avoid pregnancy for 6 months from the last flare-up. Pregnancy can activate SLE, and it may progress to severe disease either during pregnancy or in the postpartum period.

Some clinical symptoms of pregnancy can be mistaken for activation of SLE, but the presence of anti-DNA, anti-Sm, anti-Ro, and anti-La antibodies indicates activation of disease, as is a low C3 level. SLE activation during pregnancy can be difficult to differentiate from preeclampsia, as both present common symptoms such as hypertension, proteinuria, and edema.

SLE activation during pregnancy can be accompanied by fetal or maternal complications that are related to the loss of immune tolerance to the product of conception. Changes in cellular immunity, mainly in Treg cells, and activation of other immune mechanisms play an important role.

## 21. Conclusions

In pregnancy associated with SLE, immune tolerance to the semiallogeneic graft (the fetus) has to be operational in conditions of a disease with disturbed tolerance to self-antigens (SLE).

Important innate and adaptive immune changes occur. The cornerstone of these changes is represented by Treg cells.

The association of pregnancy with SLE represents a real dilemma in which the human body can either favor pregnancy or result in fetal complications (miscarriage, IUGR, preterm birth, and neonatal lupus) or maternal complications (preeclampsia; activation of SLE, mainly of lupus nephritis).

The encounter in clinical practice of pregnancy which requires immune tolerance to paternal antigens and SLE, a disease with altered tolerance to self-antigens, represents a special situation that has to be known, as it can prove useful for understanding immune tolerance mechanisms important in transplant pathology.
